# Venom Proteome of the Box Jellyfish *Chironex fleckeri*

**DOI:** 10.1371/journal.pone.0047866

**Published:** 2012-12-07

**Authors:** Diane L. Brinkman, Ammar Aziz, Alex Loukas, Jeremy Potriquet, Jamie Seymour, Jason Mulvenna

**Affiliations:** 1 Australian Institute of Marine Science, Townsville, Queensland, Australia; 2 Queensland Tropical Health Alliance, James Cook University, Queensland, Australia; 3 Queensland Emergency Medical Research Foundation, Queensland, Australia; 4 Queensland Institute of Medical Research, Brisbane, Queensland, Australia; University of New South Wales, Australia

## Abstract

The nematocyst is a complex intracellular structure unique to Cnidaria. When triggered to discharge, the nematocyst explosively releases a long spiny, tubule that delivers an often highly venomous mixture of components. The box jellyfish, *Chironex fleckeri*, produces exceptionally potent and rapid-acting venom and its stings to humans cause severe localized and systemic effects that are potentially life-threatening. In an effort to identify toxins that could be responsible for the serious health effects caused by *C. fleckeri* and related species, we used a proteomic approach to profile the protein components of *C. fleckeri* venom. Collectively, 61 proteins were identified, including toxins and proteins important for nematocyte development and nematocyst formation (nematogenesis). The most abundant toxins identified were isoforms of a taxonomically restricted family of potent cnidarian proteins. These toxins are associated with cytolytic, nociceptive, inflammatory, dermonecrotic and lethal properties and expansion of this important protein family goes some way to explaining the destructive and potentially fatal effects of *C. fleckeri* venom. Venom proteins and their post-translational modifications (PTMs) were further characterized using toxin-specific antibodies and phosphoprotein/glycoprotein-specific stains. Results indicated that glycosylation is a common PTM of the toxin family while a lack of cross-reactivity by toxin-specific antibodies infers there is significant divergence in structure and possibly function among family members. This study provides insight into the depth and diversity of protein toxins produced by harmful box jellyfish and represents the first description of a cubozoan jellyfish venom proteome.

## Introduction

Cubozoan jellyfish, commonly known as box jellyfish, are members of the Phylum Cnidaria. Cnidarians represent some of the most ancient metazoans (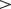
500 million years old) and their defining feature is the nematocyst (cnidocyst); a nonliving organelle housed within a specialised cell, the nematocyte (cnidocyte). The nematocyst is formed within a large post-Golgi vesicle [1] and comprises a rigid proteinaceous capsule that contains a long spiny tubule and a complex mixture of proteins (often toxins) and other small molecular weight compounds. Upon stimulation of the nematocyte's sensory receptor (cnidocil), the nematocyst discharges explosively, expelling the tubule at high speed and releasing the capsular contents [2]. A number of distinct morphological forms of nematocysts are used for a variety of purposes, including prey capture, defence or locomotory functions [3]–[5].

*C. fleckeri* is the largest and most dangerous cubozoan jellyfish to humans and its occurrence in the tropical coastal waters of Australia is a problem, particularly in summer. Nematocysts containing potent venom are prolific along the tentacles of *C. fleckeri* and cause painful and potentially life-threatening stings to humans. Symptoms of major *C. fleckeri* stings include excruciating pain, rapid acute cutaneous inflammation, dermonecrosis, permanent scarring, hypertension, hypotension, shock, dyspnoea, impaired consciousness, cardiac dysfunction and pulmonary oedema (reviewed in [6]). The onset of symptoms is extremely rapid [7] and in severe cases, death from pulmonary and/or cardiac failure can occur within minutes [8]. At least 70 deaths due to *C. fleckeri* envenoming have occurred in Australia and numerous deaths from related species have been reported in the Philippines, Maldives islands, Japan, Papua New Guinea, South India, Java, Malaysia and Gulf of Thailand [9].

Several biological activities are associated with cubozoan venoms [6]. In particular, *C. fleckeri* whole tentacle and nematocyst extracts elicit lethal, dermonecrotic, nociceptive, cytotoxic, neurotoxic, myotoxic, cardiotoxic, haemodynamic and haemolytic effects [6]. Yet, despite the medical and pharmacological significance of box jellyfish venoms to humans, their compositions have not been extensively explored. To date only two *C. fleckeri* venom proteins, CfTX-1 and -2, have been formally identified; potent haemolysins that share sequence similarity to toxins from four related cubozoan species [10], [11]. However, the broad range of bioactivities in cubozoan venoms suggests a wealth of additional venom components remain to be found.

In this work we describe the proteomic characterisaton of *C. fleckeri* venom to identify proteins that may contribute to the deleterious effects of box jellyfish stings in humans. A major challenge in the study was the paucity of nucleotide sequence coverage for *C. fleckeri* or closely related species. Although the genomes of *Hydra magnipapillata* and the sea anemone, *Nematostella vectensis*, have been described, sequences specific to cubozoans in GenBank are limited (only 74 non-redundant, non-mitochondrial protein sequences). Accordingly, in the absence of genomic or transcriptomic data, we utilised a strategy combining traditional spectral matching using Mascot with *de novo* protein sequencing from tandem mass spectrometry (MS/MS) and homology searches. Using these approaches we identified 67 proteins from the nematocysts of *C. fleckeri* including toxins and proteins involved in nematocyst and nematocyte development. We report the expansion of an important family of toxins and examine their post-translational modifications and cross-reactivity with toxin-specific antibodies. Our study represents the first venom proteome of a cubozoan jellyfish and provides insight into the depth and diversity of proteins within the unique cnidarian attribute, the nematocyst.

## Results and Discussion

### Identification of *C. fleckeri* nematocyst proteins

**Figure 1:**
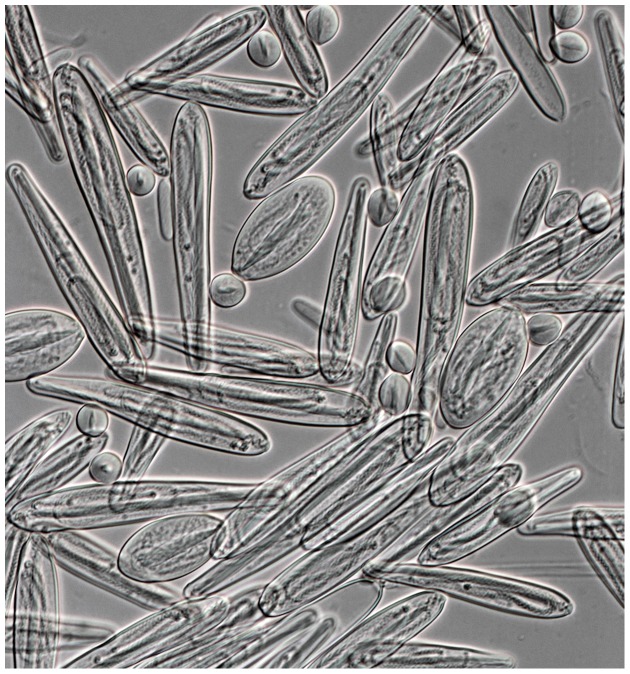
*Chironex fleckeri* nematocysts. Light microscopy image of nematocysts isolated from *C. fleckeri* tentacles (magnification 400×).

**Figure 2:**
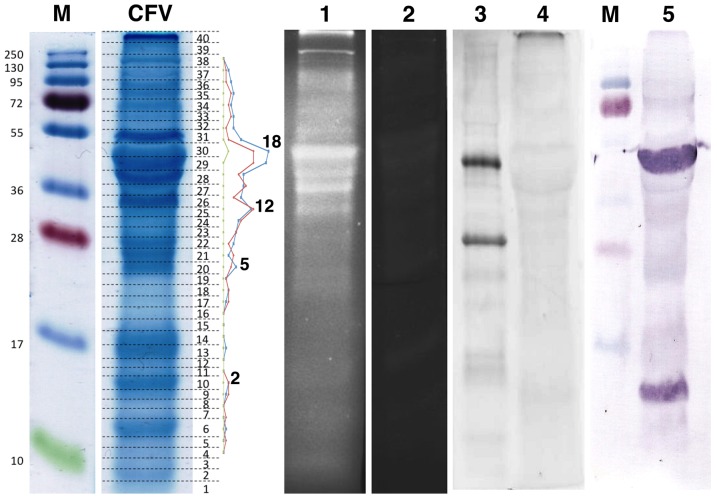
*C. fleckeri* venom proteins. Total protein from CFV in a Coomassie-stained 15% SDS-PAGE gel. Lanes were divided into 40 gel slices (dotted lines) and subjected to in-gel tryptic digest before LC-MS/MS analysis. Markers (**M**) are indicated and the numbering system used for gel slices. CFV proteins separated using SDS-PAGE and stained in-gel with fluorescent dyes reactive to glycans or phosphate groups (Lanes 1–4). Glycan analysis showed fluorescence in bands corresponding to CfTX proteins (Lane 1) and no fluorescence in the negative control (Lane 2). No phosphorylation was observed except in band 40 (Lane 4; positive control in Lane 3). Western blot analysis using polyclonal antibodies for CfTX-1 and -2 showed hybridisation in two bands corresponding to the highest scoring Mascot identifications for these proteins and in the region corresponding to approximately 12 kDa (lane 5). No other bands were positive for these proteins despite their identification in MS/MS analysis. The spectral counts for proteins from this toxin family identified using Mascot are displayed adjacent to the band numbering (blue, red and green lines for CfTX-1, CfTX-2 and CqTX-A resp.). Actual spectral counts for a selection of CfTX-1 points are shown for reference.

**Figure 3:**
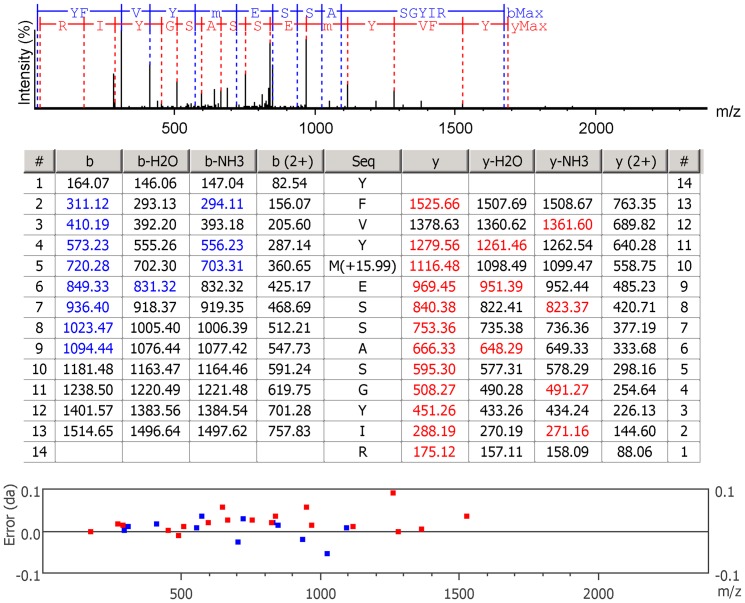
Representative example of a *de novo* sequence match to CaTX-A using a homology search. Representative spectra from a *de novo* match to the toxin protein CaTX-A. The top panel shows the spectrum annotated with the y and b-ion series, the middle panel shows fragment ions detected and the bottom panel the errors, in daltons, for each fragment ion.

*C. fleckeri* venom (CFV) was purified from nematocysts purified in a discontinuous Percoll gradient (Figure 1). Prior to MS/MS analysis, the CFV was separated by SDS-PAGE (Figure 2) and in-gel tryptic digests were performed on 40 gel fragments. In addition to SDS-PAGE, tryptic peptides from total CFV were subjected to OFFGEL electrophoresis (OGE). There are only 21 *C. fleckeri* protein sequences in GenBank and only 186 for Cubozoa (as of 13th August, 2012). Accordingly, a strategy incorporating both spectral searches using Mascot, with false discovery analysis conducted using X! Tandem and Scaffold, was combined with *de novo* analysis of generated spectra using PEAKS. Two different PEAKS searches were conducted, the first, analogous to a Mascot search, used only exact peptide matches derived from high quality *de novo* sequence tags. This was used to confirm and extend Mascot searches. The second search allowed for matching of high quality *de novo* sequence tags to homologous sequences in the target database, thus permitting the detection of proteins with similar sequence (see Figure 3 for a representative spectra). Both PEAKS search strategies utilised decoy databases to derive false positive rates. Following these searches, 46 proteins were identified using Mascot and PEAKS at a 0% FDR for Mascot identifications and a 1% FDR for PEAKS (Table 1, Information S1, S2 and S5). *De novo* homology searches conducted using PEAKS resulted in the identification of 46 proteins at a FDR of 1%, 16 of which had not been identified in the first search (Table 2, Information S3, S4, S5). Nine of these were single peptide identifications and annotated spectra for these are provided in Information S6. In total, sixty-one non-redundant protein identifications were made at a high level of significance using the two search strategies.

**Table 1:** Protein identifications using Mascot and PEAKS.

		Scaffold				Peaks		SP	H
ID	Description	USC	CO	−10lgP	SC	USC	CO		
**Toxins**									
160380600	Toxin CfTX-1	26	41	359.39	42	21	54	+	+
160380601	Toxin CfTX-2	16	43	327.90	31	23	63	+	+
18146993	Toxin CqTX-A	2	23	224.45	16	1	27	+	+
**Nematogalectins**									
219233028	Nematogalectin-related	-	-	148.12	3	1	14	+	+
156401683	Nematogalectin-related	5	17	124.05	4	4	12	+	+
308210726	Nematogalectin	-	-	93.89	2	1	14	+	+
**Structural**									
159254	Actin	12	38	190.23	7	1	28	−	+
26522784	Actin	-	-	194.30	9	1	23	−	+
282471169	Actin	-	-	137.66	3	1	16	−	+
162122963	Actin	-	-	108.89	3	1	22	−	+
294372631	Non-muscle actin	-	-	158.11	4	1	25	−	+
156223974	Alpha tubulin	16	44	77.48	4	1	12	−	+
221129327	Beta tubulin	16	39	206.15	15	3	45	−	+
221129323	Beta tubulin	-	-	129.01	5	1	11	−	+
156369515	Tubulin beta-2C chain	-	-	89.33	3	3	14	−	+
225547767	Clathrin heavy chain	2	7	-	-	-	-	−	−
221130531	Clathrin heavy chain 1	2	2	124.69	3	3	2	−	−
221107857	Histone H2B-1	-	-	69.50	2	1	23	−	+
156219637	Histone H2B	-	-	126.82	6	1	30	−	+
156219675	Histone H3.2-like	-	-	81.04	3	2	11	−	+
156198376	Histone H2A	2	12	137.98	7	3	36	−	+
163678147	Histone 2	-	-	62.91	2	1	15	−	+
**Heat shock proteins**									
221132017	Heat shock protein	3	4	88.74	2	1	10	+	−
262478028	90 heat-shock protein	2	12	-	-	-	-	−	+
**Oxido-reductive**									
100913623	Cytochrome c oxidase subunit II	2	11	-	-	-	-	−	−
156364605	ATP synthase beta subunit	2	10	-	-	-	-	−	+
156213313	ATP synthase	5	18	-	-	-	-	−	+
162106843	ATP synthase subunit alpha	-	-	103.04	3	3	13	−	+
82862750	V-type proton ATPase	-	-	52.02	2	1	20	−	+
221111162	Thioredoxin peroxidase	2	10	-	-	-	-	−	+
**Dickkopf**									
221115495	Dickkopf protein 3	4	38	216.04	11	9	38	+	+
37498690	Dickkopf-3 related protein	5	27	237.58	12	11	53	+	+
**Proteases**									
221116853	Neprilysin	3	4	116.69	2	1	3	+	+
221128287	Neprilysin	-	-	102.93	2	1	3	+	+
221132105	Endothelin-converting enzyme 2	-	-	85.94	2	2	3	+	+
156386196	Endothelin-converting enzyme 2-like	-	-	56.17	2	2	3	+	+
**Miscellaneous**									
221118656	DNA-directed RNA polymerase II	2	1	189.76	9	9	3	+	+
156223696	Ras-related protein Rab-35	2	12	-	-	-	-	−	−
239736186	Elongation factor-1 alpha	8	22	64.17	2	2	5	−	−
221129496	DNA-directed RNA polymerase, omega	-	-	120.92	2	2	1	−	+
221119922	Gamma-glutamyltranspeptidase 1	-	-	66.25	2	2	2	+	+
221120305	Gamma-glutamyl hydrolase	-	-	67.12	2	2	3	−	+
**Unknown**									
221123368	Hypothetical protein [Hydra magnipapillata]	2	2	135.12	4	4	3	+	−
221113955	Hypothetical protein [Hydra magnipapillata]	2	14	124.91	2	2	14	+	−
221116631	Predicted protein [Hydra magnipapillata]	2	1	107.93	2	2	1	−	−

Protein identifications using Mascot and PEAKS. Abbreviations used: ID — identification number corresponding to custom database supplied as S5; USC — number of unique and significant spectra contributing to the identification; SC — total number of significant spectra contributing to the identification; CO — percent cover; and −10lgP — the PEAKS probability based score. A ‘+’ in the SP column denotes the presence of a signal sequence and a ‘+’ in the ‘H’ column denotes the identification of a similar protein in the *H. magnipapillata* venom proteome.

**Table 2:** Additional proteins identified using a homology search of *de novo* sequence tags in PEAKS.

ID	−10lgP	CO	SC	USC	Description	SP	H
**Toxins**							
20137964	45.69	3	1	1	Toxin CaTX-A [Carybdea alata]	+	+
20137965	56.47	4	1	1	Toxin CrTX-A [Carybdea rastonii]	+	+
260066272	46.78	2	1	1	Cytotoxin A isoform 1 [Malo kingi]	−	+
**Nematogalectins**							
306408168	93.89	13	2	1	Nematogalectin B [Hydra magnipapillata]	+	+
**Structural**							
221107145	90.25	10	2	2	Collagen alpha-1(XII) chain [Hydra magnipapillata]	+	+
156388143	61.02	3	1	1	Collagen alpha-2(IX) chain [Nematostella vectensis]	−	+
221121786	54.27	1	1	1	Procollagen type XIV alpha 1 [Hydra magnipapillata]	−	+
**Proteases**							
156400736	54.28	8	2	2	Carboxypeptidase [Nematostella vectensis]	−	+
156226146	39.08	3	1	1	Carboxypeptidase D [Nematostella vectensis]	−	+
**Miscellaneous**							
156401103	72.46	8	2	2	ADP,ATP carrier protein-like [Nematostella vectensis]	−	−
221112215	59.92	2	1	1	Gamma-glutamyl transferase 1a [Hydra magnipapillata]	−	+
221116631	119.78	1	3	3	Viral A-type inclusion protein	−	−
219231428	53.83	6	1	1	Tyrosine kinase receptor [Anemonia viridis]	−	−
223649562	34.95	5	1	1	Nematoblast-specific protein nb012a [Hydra oligactis]	+	−
**Unknown**							
221116853	116.69	3	2	1	Predicted protein [Hydra magnipapillata]	+	−
50698431	60.51	17	2	2	Hypothetical protein [Hydractinia echinata]	−	−

In total 46 proteins were identified in homology searches, 17 of which were not identified in the Mascot and PEAKS searches. Abbreviations used: ID — identification number corresponding to custom database supplied as S5; −10lgP — PEAKS probability score; CO — percent cover; SC — total number of significant spectra contributing to the identification; USC — number of unique and significant spectra contributing to the identification. A ‘+’ in the SP column denotes the presence of a predicted signal sequence using SignalP and a ‘+’ in the ‘H’ column denotes the identification of a similar protein in the *H. magnipapillata* venom proteome. For complete list of proteins identified in *de novo* searches see Information S3.

### Overall composition of CFV proteins

When categorised into functional groupings (Table 1 and 2) the most abundantly identified proteins reflect the known composition of the nematocyst, with toxins, collagens, dickkopf-3 proteins and nematogalectins all identified. The most commonly identified proteins were structural in nature, reflecting the composition of the nematocyst capsule, which is primarily composed of mini-collagens [5], and the tubule in which nematogalectin is a major component [12]. The detection of numerous structural proteins in *C. fleckeri* venom can be explained by the chemical method used for venom extraction. Dithiothreitol, a strong reducing agent, was used to partially disintegrate the nematocyst capsule and cause venom release, so it is likely that a proportion of capsular components and other structural proteins were solubilised during this process. In *Hydra*, a number of genes have been found to be specifically expressed in the nematocyte, including those encoding for toxins, proteins involved in the assembly of the nematocyst capsule, tubule and spines, as well as proteins associated with the cnidocil of the nematocyte [13], [14]. A variety of these proteins were also identified in CFV, including tubulin, 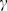-glutamyltransferase and the nematogalectins. As found in proteomic studies of *Hydra* [15], the proteins identified here are a mixture of secreted and non-secreted proteins with only 38% containing a classical secretory signal peptides targeting to the general secretory pathway. Many of these non-secreted proteins were likely present during nematocyst formation and do not have a specific function in the venom, although they may play an important role in nematocyst formation. Five proteins were identified that had no homology to proteins outside Cnidaria, three of which contained signal sequences, suggesting that some of the known bioactivities of CFV may be mediated by proteins unique to the phylum. A comparison of the protein families identified in this work with the recently published *H. magnipapillata* venom proteome showed that 75% of the proteins identified in the CFV were also present in the *Hydra* nematocyst.

### *C. fleckeri* toxin proteins and isoforms

**Figure 4:**
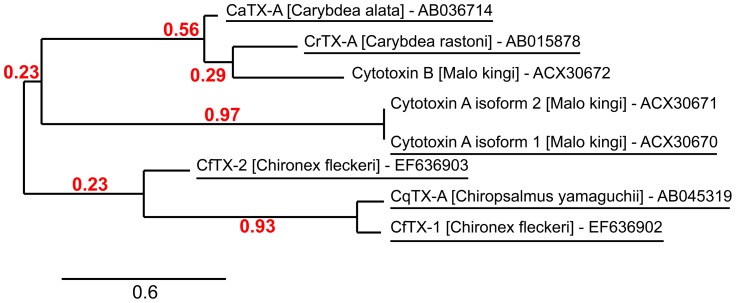
Phylogenetic tree of Cubozoan toxin proteins. Phylogenetic tree depicting the grouping of box jellyfish toxins found in GenBank into three broad classes. Proteins identified in this study are underlined and their GenBank accession number is indicated. The tree was produced using MUSCLE and PhyML for tree building and the aLRT statistical test [50] was used for branch support.

A large number of toxic effects are attributed to *C. fleckeri* venom, but only two *C. fleckeri* toxins, CfTX-1 and -2, have been identified through peptide and cDNA sequencing [10], [11]. These proteins are related to box jellyfish toxins CrTX-A, CaTX-A and CqTX-A isolated from the venoms of *Carybdea rastonii* [16], *Carybdea alata* [17] and *Chironex yamaguchii* [18] (as Chiropsalmus quadrigatus; renamed 2009 [19]), respectively, as well as Cytotoxin A (isoforms 1 and 2) and Cytotoxin B (partial sequence), retrieved from a *Malo kingi* tentacle cDNA library [20] (see also [21] for disputed species identification). Using Mascot, three members of this family were identified, CfTX-1 and -2 and a homologue of CqTX-A (Table 1. A further three homologues of CaTX-A, CrTX-A and Cytotoxin A isoform 1, from *M. kingi*, were identified on the basis of homology (Table 2). Members of this toxin family are potently haemolytic and cause pain, inflammation, dermonecrosis and death in experimental animals [10], [11], [16]–[18], suggesting the toxins play an important functional role in box jellyfish envenoming. Computational analyses of the toxin sequences point to a pore-forming mechanism of action due to predictions of common transmembrane spanning regions and weak structural similarities to pore-forming insecticidal 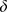-endotoxins [6]. Recently, proteins with sequence homology to the box jellyfish toxins have also been identified in the venoms of *Cyanea capillata* (Scyphozoa) [22] and *H. magnipapillata* (Hydrozoa) [15] inferring the toxin family is present throughout Cnidaria. Construction of a phylogenetic tree of currently available cubozoan protein sequences shows three groupings within the toxin family, suggesting structural and fn diversification has occurred between toxin groups during evolution. (Figure 4).

Both Mascot and *de novo* homology searches suggest that additional isoforms of the CfTX toxins are present in CFV. CfTX-1 and -2 were identified using Mascot in 29 of the 40 gel bands analysed yet the presence of well defined bands on SDS-PAGE suggests this was not the result of protein breakdown (Figure 2). This was supported by the identification of CqTX-A and the identification, on the basis of homology, of a further three examples of the toxins. Western blot analysis using polyclonal antibodies against CfTX-1 and -2 showed hybridisation to one major band (spanning gel bands 28 & 29) that provided the highest scoring Mascot identifications to CfTX-1 and -2 as well as a band, possibly a cleavage product, in the lower molecular weight region of the gel (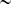
12 kDa; gel band 10) (Figure 2). No other bands reacted positively towards the antibodies, suggesting that although a number of CfTX-like proteins are present in *C. fleckeri* venom, they do not contain common epitopes to which CfTX-1 and -2 specific antibodies can bind. Sequence divergence among toxin family members coupled with a lack of cross-reactivity by toxin-specific antibodies suggests that there are significant structural variations between the related toxins that could modulate their function and/or specificity. Although these toxins appear to be the major CFV toxin family, two isoforms of both neprilysin and endothelin-converting enzymes where also identified. Neprilysin, a metallo-endopeptidase, has been identified as a possible neurotoxic protein in snake venom [23] while the endothelin-converting enzymes may play a supporting role, as is the case in wasp venom [24], by processing pro-proteins into mature protein or peptide toxins.

### Proteins involved in nematocyst structure and nematogenesis

Proteins identified in the *Hydra* nematocyst capsule are predominantly comprised of different species of mini-collagens that form a disulfide-linked polymer [25]. In this study no collagen was identified using Mascot, although three collagen isoforms were identified on the basis of homology. Given the limitations of available sequence for proteomic analysis, it is likely that sequence divergence between characterised cnidarians and *C. fleckeri* collagens resulted in fewer identifications in this study; the presence, in homology searches, of multiple collagen identifications at a level of significance below that permitted in the study supports this conjecture. Likewise, NOWA, another major protein constituent of the nematocyst capsule, was also identified at a lower significance level, suggesting that the protein is found in CFV but that its sequence has diverged from those NOWA sequences currently in the database. Multiple isoforms of nematogalectin, the main protein constituent of the tubule, were identified in CFV, suggesting that, like *Hydra* but unlike the anthozoans, *C. fleckeri* contains two copies of this gene [12], [26]. Spinalin, a spine protein, was not identified at any level of significance but is more resistant to dissolution in the presence of DTT than other structural nematocyst proteins [27].

The explosive potential of the nematocyst is due to an exceptionally high intracapsular pressure produced by a high concentration of poly-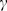-glutamate (pG) that binds a 2M concentration of cations [28]. A key enzyme in the production of pG, 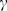-glutamyltransferase, was identified in CFV confirming previous studies that showed enzymes necessary for pG synthesis are transferred into the capsule prior to the hardening of the capsule wall [29], [30]. Poly-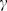-glutamate degradative activity has been reported in *Hydra* [31] and a 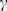-glutamyl hydrolase was identified in CFV. This molecule catalyses the hydrolysis of 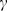-glutamyl bonds and may indicate that some regulation of pG biosynthesis occurs in the developing nematocyst. Similar to the *Hydra* nematocyst proteome, several isoforms of the nematogenesis-associated dickkopf protein 3 (Dkk3) [32], [33] were identified. Dkk3 belongs to the dickkopf family of developmental proteins that purportedly act via inhibition of the Wnt signalling pathway [33] and its presence in CFV suggests that nematogenesis in *C. fleckeri* proceeds along similar lines to other better characterised cnidarians.

### Post-translation modifications

Both nematogalectins [12] and NOWA [34] are known to be glycosylated, and in snake venom the glycosylation of key venom proteins is thought to enhance protein stability and diffusion [35]. *In silico* analysis of the primary sequences of toxins CfTX-1 and -2 showed a number of conserved motifs, indicative of a range of post-translation modifications including phosphorylation and glycosylation. Therefore, to test for these modifications, total CFV was stained with fluorescent dyes after SDS-PAGE. For glycan staining, intense fluorescence was observed in bands confirmed to contain CfTX-1 and -2 by tandem MS (Figure 2; band 30); weaker staining was observed in areas corresponding to the identification of the nematogalectins (bands 34 and 26 and 27 respectively). Further staining was observed in areas thought to contain CfTX isoforms suggesting that glycosylation is a common post-translational modification of the *C. fleckeri* toxin proteins. Phosphorylation analysis indicated that no CFV proteins were phosphorylated, with the exception of a protein in band 40 which was identified as 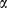-tubulin. In comparison, phosphorylation is common in snake venoms but restricted to glycoproteins containing phosphorylated carbohydrates [35]. Glycosylated carbohydrate moieties are common in nature and are often involved in lysosomal targeting by way of mannose 6-phosphate receptors [36] and their absence here suggests this pathway is not utilised during venom production in the nematocytes.

## Conclusions

The principle aim of this study was to identify proteins in the venom of *C. fleckeri* that could be responsible for its wide range of bioactivities and debilitating effects in humans. As our results show, the venom proteome of *C. fleckeri* contains a diverse array of proteins dominated by toxins and proteins involved in nematogenesis. The most abundant toxins in *C. fleckeri* venom are CfTX-1, CfTX-2 and a number of newly discovered CfTX-like isoforms. Toxicological data and bioinformatic information suggest that this expanding toxin family plays a significant functional role in envenoming and further research is necessary to elucidate the actions of these toxins at the molecular level. Due to the lack of genomic and transcriptomic sequences currently available for *C. fleckeri*, or closely related species, it is also likely that other toxins unique to Cubozoa are yet to be discovered.

The CfTX-like proteins belong to a family of potent toxins that is only found in Cnidaria. The evolutionary emergence of a particular protein family in venoms is a consistent theme in nature, with examples including the conotoxins, from cone snails [37], and the Ancylostoma secreted proteins from hookworms [38]. All of these organisms are currently being utilised as a source of therapeutic compounds [37], [39] and the expansion of the cnidarian toxin family promises to provide a rich source of novel bioactive compounds that could be utilised as prospective new drugs, novel research tools or other beneficial purposes. Finally, gaining a better understanding of the molecular, structural and functional diversity of this important toxin family will lead to the better understanding of and improvements in medical treatments of cnidarian stings.

## Materials and Methods

### Nematocyst Isolation

Nematocysts were isolated from excised tentacles [40] of a mature specimen (captured near Weipa, Queensland, Australia), except the nematocysts were not lyophilised. Isolated nematocysts were further purified in a discontinuous Percoll gradient [11]. The integrity of the undischarged nematocysts was verified using an Axioskop2 mot plus light microscope (Zeiss). No specific permits were required for the described field studies. No specific permissions were required as the animals collected are not protected and were collected from marine environments that are not protected or privately owned. *C. fleckeri* is not an endangered or protected species.

### Electrophoresis and In-gel Digestion

Percoll-cleaned nematocysts were washed twice with 20 mM Tris-HCl (pH 7.5). Aliquots of nematocysts were resuspended 1∶6 (wet w/v) in reducing SDS-sample buffer [41] containing DTT and incubated at RT until 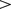
90% nematocyst discharge was observed microscopically. The samples were centrifuged (16 k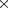 g, 4°C, 10 min) to remove capsular debris and supernatants were transferred to clean tubes and heated (95°C, 5 min). Duplicate samples (10 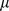
L) were applied to a 15% SDS-PAGE gel and electrophoresis performed according to Laemmli [41]. Proteins were stained with EZBlue G-250 colloidal Coomassie stain (Sigma) and each sample lane was divided into 40 gel slices. In-gel trypsin digestion was performed using established methods [42] and after digestion peptide mixtures were reduced to 12 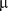
l in a vacuum centrifuge before mass spectral analysis.

### OFFGEL electrophoresis

Established methods [42] were used to reduce, alkylate and trypsinize two 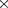
2.5 mg samples of total protein from lyophilised *C. fleckeri* venom extracted from nematocysts using bead mill homogenisation. The resulting tryptic fragments were subjected to OFFGEL electrophoresis (OGE). The 3100 OFFGEL Fractionator and OFFGEL Kit pH 3–10 (Agilent Technologies) with a 24-well setup were prepared as per the manufacturers protocols. The tryptic digests were diluted in peptide-focusing buffer, without the addition of ampholytes, to a final volume of 3.6 ml and 150 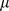
l was loaded into each well. The samples were focused with a maximum current of 50 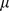
A until 50 kVh were achieved. Peptide fractions were harvested, lyophilised and resuspended in 5% formic acid before LC and mass spectral analysis.

### Western Blot Analysis

Polyclonal antibodies against CfTX-1 and -2 were commercially obtained from IMVS, Veterinary Services (Gilles Plains, Australia) under the aegis of the IMVS Animal Ethics Committee (license number 155) as previously described [11]. Venom proteins were separated by SDS-PAGE as described above and transferred to Immobilon-P PVDF membrane (Millipore). The membrane was blocked (5% (w/v) skim milk powder in TBST, 0.5 h) and incubated overnight with the rabbit antibodies diluted in blocking solution (1∶2000). The membrane was washed (3×10 min in TBST) then incubated (1 h) with goat anti-rabbit alkaline phosphatase-conjugated antibodies (Sigma) diluted in TBST (1∶5000). Following membrane washing, antibody-bound proteins were visualised using NBT/BCIP (Promega).

### Glycoprotein and Phosphoprotein Staining

Following separation of nematocyst-derived proteins by SDS-PAGE, in-gel detection of glycoproteins and phosphoproteins was performed using a GlycoProfile III fluorescent glycoprotein detection kit (Sigma) or Pro-Q Diamond phosphoprotein gel stain (Invitrogen), respectively, according to the manufacturers' instructions. For glycoprotein analysis, a duplicate gel was processed omitting the oxidation step to detect any non-specific fluorescent staining. For phosphoprotein analysis, the ProteoProfile PTM marker (Sigma) containing phosphorylated ovalbumin (45 kDa) and 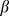-casein (30 kDa) was included as a positive control. Fluorescently stained glycoproteins and phosphoproteins were visualised using a ChemiSmart 3000 image acquisition system (Viber Lourmat).

### Protein Identification using MS/MS

OGE fractions and tryptic fragments from in-gel digests were chromatographically separated on a Dionex Ultimate 3000 HPLC using an Agilent Zorbax 300SB-C18 (3.5 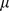
m, 150 mm×75 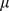
m) column and a linear gradient of 0–80% solvent B over 60 min. A flow rate of 300 nl/min was used for all experiments. The mobile phase consisted of solvent A (0.1% formic acid (aq)) and solvent B (80/20 acetonitrile/0.1% formic acid (aq)). Eluates from the RP-HPLC column were directly introduced into the NanoSpray II ionisation source of a QSTAR Elite Hybrid MS/MS System (Applied Biosystems) operated in positive ion electrospray mode. All analyses were performed using Information Dependant Acquisition. Analyst 2.0 (Applied Biosystems) was used for data analysis and peak list generation. Briefly, the acquisition protocol consisted of the use of an Enhanced Mass Spectrum scan as the survey scan. The three most abundant ions detected over the background threshold were subjected to examination using an Enhanced Resolution scan to confirm the charge state of the multiply charged ions. The ions with a charge state of 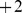, 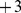 or with unknown charge were then subjected to collision-induced dissociation using a rolling collision energy dependent upon the m/z and the charge state of the ion. Enhanced Product Ion scans were acquired resulting in full product ion spectra for each of the selected precursors which were then used in subsequent database searches.

### Mascot searches

Searches were performed using version 2.2.02 of Mascot with a 0.1 Da tolerance on the precursor, 0.1 Da tolerance on the product ions, allowing for methionine oxidation and carbamidomethylation as fixed and variable modifications respectively, two missed cleavages, charge states +2 and +3, trypsin as the enzyme and MudPIT scoring was used to derive protein scores. All experiments were searched against a custom-built protein database of 160,848 proteins comprised of protein sequences derived from all cnidarian nucleotide sequence in the NCBI non-redundant (nr) database. Nucleotide sequences were cleaned using SeqClean (http://compbio.dfci.harvard.edu/tgi/software/) in conjunction with the UniVec vector sequence database from the NCBI. Sequences were then clustered using CAP3 [43] and a set of predicted protein sequences generated using ESTScan [44]. Searches were also made against the SwissProt database to detect contamination. Mascot searches were further validated using Scaffold (version Scaffold_3_00_06, Proteome Software Inc). Using Scaffold X! Tandem searches were performed on a subset of the custom database using the same parameters as used for Mascot searches. Peptide identifications were accepted if they could be established at greater than 95.0% probability as specified by the PeptideProphet algorithm [45]. Protein identifications were accepted if they could be established at greater than 95.0% probability and contained at least two identified peptides. Protein probabilities were assigned by the ProteinProphet algorithm [46]. Proteins that contained similar peptides and could not be differentiated based on MS/MS analysis alone were grouped to satisfy the principles of parsimony. A 0.0% false discovery rate (FDR) was calculated using Scaffold validated protein identifications.

### PEAKS and *de novo* sequencing

*De novo* protein sequencing was achieved using PEAKS (version 4.5, Bioinformatics Solutions, Waterloo, Canada) [47]. *De novo* peptide sequences were derived from the combined MS/MS spectra from in-gel digests using 0.1 Da tolerance on the parent and fragment ions, digestion with trypsin, one missed cleavage and allowing for methionine oxidation and carbamidomethylation as fixed and variable modifications respectively. The *de novo* tags where then used in two searches, using PEAKS, of the custom database performed using the same parameters. The first utilised high quality *de novo* sequences to identify exactly matching peptides from the custom database. A decoy database was searched and spurious identifications were used to calculate a FDR for peptide identifications using the decoy-fusion method [47]. Using a desired false discovery rate of 1%, proteins were accepted only if they possessed at least two peptides scoring above the cutoff calculated for the desired FDR and if at least one significant peptide was unique to that protein. PEAKS was also used to conduct homology searches using the *de novo* sequence tags. In this search, the amino acid sequence of the *de novo* tags was used to search for homologous peptides, rather than exact matches, in the custom database. The same FDR procedure was used to estimate a peptide cutoff score and protein identifications were accepted only if they contained at least one unique and significant peptide. Identifications from homology searches containing only a single peptide were manually annotated and only high quality identifications, containing the majority of calculated fragment ions, were accepted.

### Bioinformatic analysis

Protein descriptions were assigned to protein identifications using BLASTP on the non-redundant protein databases from NCBI (bit score 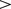
30). Classical secretory signal sequences were detected using a local version of SignalP [48]. The phylogenetic tree was produced using MUSCLE for multiple alignment, Gblocks for automatic alignment curation, PhyML for tree building and TreeDyn for tree drawing using the tree-generation pipeline at Phylogeny.fr website [49]. The aLRT statistical test [50] was used for branch support.

## Supporting Information

Information S1 **Scaffold peptide and protein reports.** Excel spreadsheet containing full protein and peptide information for *C. fleckeri* Mascot identifications.
(XLS)

Information S2 **Peaks peptide report.** Excel spreadsheet containing full peptide information for *C. fleckeri* PEAKS identifications.
(XLSX)

Information S3 **Proteins identified during homology searches.** Proteins identified using a homology search of de novo sequence tags in PEAKS. Abbreviations used: ID — identification number corresponding to custom database supplied as S5; −10lgP — PEAKS probability score; CO — percent cover; SC — total number of significant spectra contributing to the identification; USC — number of unique and significant spectra contributing to the identification. A ‘+’ in the SignalP column denotes the presence of a predicted signal sequence using SignalP and a ‘+’ in the Hydra column denotes the identification of a similar protein in the *H. magnipapillata* venom proteome.
(XLSX)

Information S4 **Homology peptide report.** Excel spreadsheet containing full peptide information for *C. fleckeri* identifications made during homology searches of de novo peptide sequences.
(XLSX)

Information S5 **Sequences of identified proteins.** Sequences of proteins identified during Mascot and PEAKS searches in fasta format.
(FASTA)

Information S6 **Annotated single peptide identifications.** Annotated spectra, ion tables and error plots of all proteins identified by a single peptide during homology searches using PEAKS.
(PDF)
