# Supplementary material for: Venom Proteome of the Box Jellyfish Chironex fleckeri
Source: PLoS One. 2012 Dec 7;7(12):e47866. doi: 10.1371/journal.pone.0047866 (PMC3517583; doi:10.1371/journal.pone.0047866)

20137964

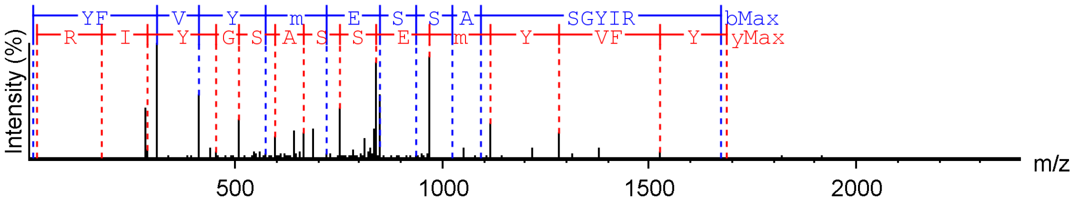

#	b	b-H2O	b-NH3	b (2+)	Seq	y	y-H2O	y-NH3	y (2+)	#
1	164.07	146.06	147.04	82.54	Y					14
2	311.12	293.13	294.11	156.07	F	1525.66	1507.69	1508.67	763.35	13
3	410.19	392.20	393.18	205.60	V	1378.63	1360.62	1361.60	689.82	12
4	573.23	555.26	556.23	287.14	Y	1279.56	1261.46	1262.54	640.28	11
5	720.28	702.30	703.31	360.65	M(+15.99)	1116.48	1098.49	1099.47	558.75	10
6	849.33	831.32	832.32	425.17	E	969.45	951.39	952.44	485.23	9
7	936.40	918.37	919.35	468.69	S	840.38	822.41	823.37	420.71	8
8	1023.47	1005.40	1006.39	512.21	S	753.36	735.38	736.36	377.19	7
9	1094.44	1076.44	1077.42	547.73	A	666.33	648.29	649.33	333.68	6
10	1181.48	1163.47	1164.46	591.24	S	595.30	577.31	578.29	298.16	5
11	1238.50	1220.49	1221.48	619.75	G	508.27	490.28	491.27	254.64	4
12	1401.57	1383.56	1384.54	701.28	Y	451.26	433.26	434.24	226.13	3
13	1514.65	1496.64	1497.62	757.83	I	288.19	270.19	271.16	144.60	2
14					R	175.12	157.11	158.09	88.06	1

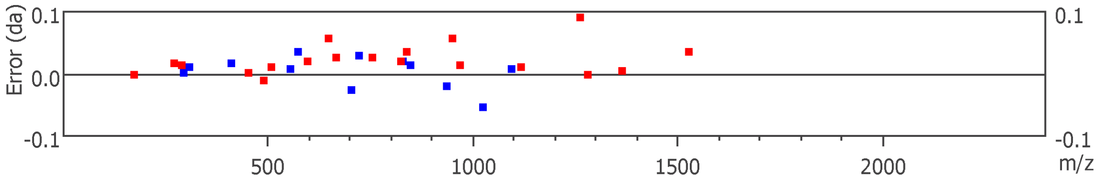

20137965

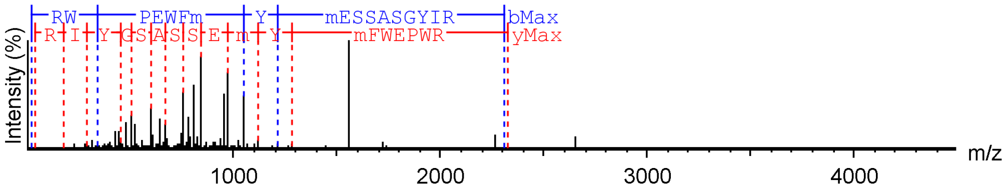

#	b	b-H2O	b-NH3	b (2+)	Seq	y	y-H2O	y-NH3	y (2+)	#
1	157.11	139.10	140.08	79.05	R					18
2	343.16	325.18	326.16	172.09	W	2171.92	2153.91	2154.89	1086.46	17
3	440.24	422.23	423.18	220.62	P	1985.84	1967.83	1968.81	993.42	16
4	569.28	551.27	552.26	285.14	E	1888.79	1870.78	1871.76	944.89	15
5	755.36	737.35	738.34	378.18	W	1759.75	1741.73	1742.72	880.37	14
6	902.43	884.42	885.40	451.72	F	1573.67	1555.66	1556.64	787.33	13
7	1049.52	1031.46	1032.44	525.26	M(+15.99)	1426.60	1408.59	1409.57	713.80	12
8	1212.54	1194.52	1195.52	606.78	Y	1279.66	1261.55	1262.54	640.28	11
9	1359.57	1341.53	1342.54	680.29	M(+15.99)	1116.47	1098.47	1099.47	558.73	10
10	1488.61	1470.60	1471.58	744.80	E	969.44	951.45	952.44	485.23	9
11	1575.64	1557.63	1558.61	788.33	S	840.40	822.38	823.39	420.71	8
12	1662.67	1644.66	1645.65	831.84	S	753.37	735.38	736.33	377.19	7
13	1733.71	1715.70	1716.68	867.35	A	666.34	648.35	649.26	333.68	6
14	1820.74	1802.73	1803.71	910.87	S	595.30	577.31	578.28	298.14	5
15	1877.76	1859.75	1860.74	939.45	G	508.27	490.28	491.26	254.64	4
16	2040.83	2022.82	2023.80	1020.91	Y	451.26	433.26	434.20	226.13	3
17	2153.91	2135.90	2136.88	1077.46	I	288.19	270.19	271.17	144.60	2
18					R	175.11	157.11	158.09	88.06	1

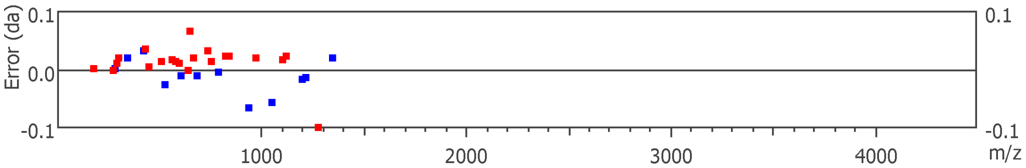

260066272

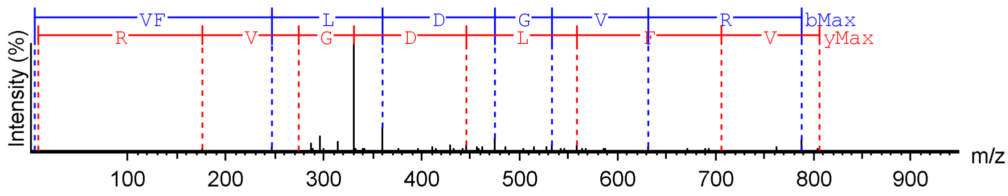

#	b	b-H2O	b-NH3	b (2+)	Seq	y	y-H2O	y-NH3	y (2+)	#
1	100.08	82.07	83.05	50.54	V					7
2	247.11	229.13	230.12	124.07	F	706.36	688.38	689.33	353.69	6
3	360.19	342.17	343.20	180.61	L	559.30	541.31	542.26	280.16	5
4	475.21	457.22	458.17	238.13	D	446.25	428.23	429.22	223.62	4
5	532.21	514.24	515.25	266.64	G	331.20	313.20	314.19	166.10	3
6	631.37	613.33	614.36	316.17	V	274.16	256.18	257.15	137.59	2
7					R	175.12	157.11	158.10	88.06	1

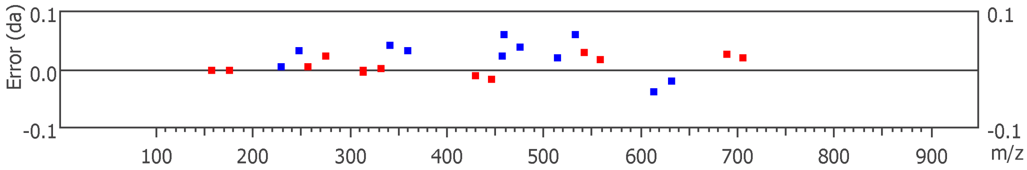

156388143

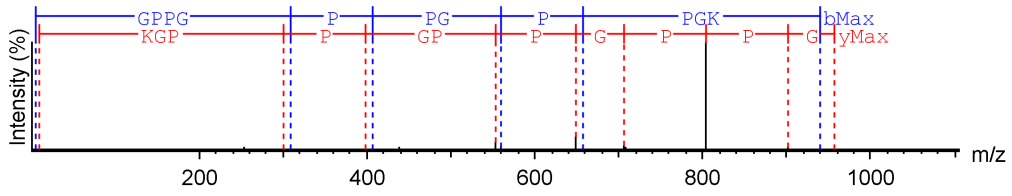

#	b	b-H2O	b-NH3	b (2+)	Seq	y	y-H2O	y-NH3	y (2+)	#
1	58.03	40.02	41.00	29.51	G					11
2	155.08	137.07	138.06	78.04	P	900.54	882.48	883.47	450.75	10
3	252.13	234.12	235.11	126.57	P	803.44	785.41	786.41	402.22	9
4	309.15	291.15	292.13	155.08	G	706.39	688.38	689.36	353.69	8
5	406.22	388.20	389.18	203.60	P	649.36	631.36	632.34	325.18	7
6	503.26	485.25	486.23	252.13	P	552.28	534.30	535.29	276.66	6
7	560.23	542.27	543.26	280.64	G	455.26	437.20	438.23	228.13	5
8	657.33	639.26	640.31	329.17	P	398.20	380.23	381.21	199.62	4
9	754.39	736.38	737.36	377.69	P	301.19	283.18	284.16	151.09	3
10	811.41	793.40	794.38	406.21	G	204.13	186.12	187.11	102.57	2
11					K	147.11	129.10	130.09	74.06	1

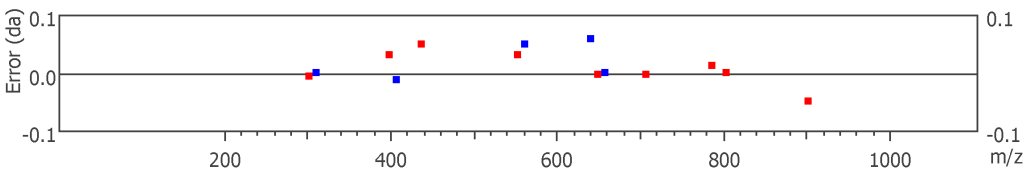

221121786

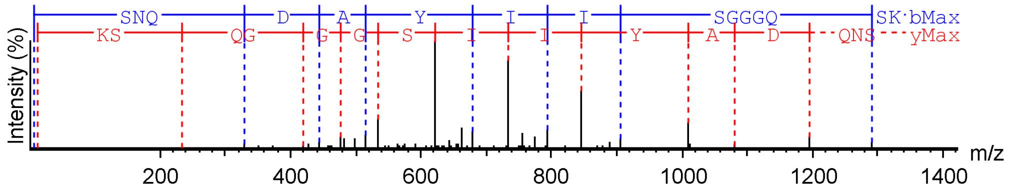

#	b	b-H2O	b-NH3	b (2+)	Seq	y	y-H2O	y-NH3	y (2+)	#
1	88.04	70.03	71.01	44.52	S					15
2	202.08	184.07	185.06	101.54	N	1437.70	1419.69	1420.67	719.35	14
3	330.12	312.14	313.11	165.57	Q	1323.65	1305.64	1306.63	662.33	13
4	445.12	427.13	428.14	223.08	D	1195.55	1177.58	1178.57	598.30	12
5	516.19	498.16	499.14	258.60	A	1080.59	1062.56	1063.54	540.78	11
6	679.27	661.26	662.24	340.13	Y	1009.47	991.52	992.50	505.27	10
7	792.36	774.29	775.34	396.68	I	846.46	828.46	829.39	423.73	9
8	905.44	887.43	888.48	453.22	I	733.35	715.38	716.36	367.19	8
9	992.47	974.46	975.44	496.73	S	620.27	602.29	603.27	310.65	7
10	1049.49	1031.48	1032.46	525.25	G	533.23	515.26	516.19	267.13	6
11	1106.51	1088.50	1089.48	553.76	G	476.21	458.24	459.22	238.62	5
12	1163.53	1145.52	1146.51	582.27	G	419.23	401.21	402.20	210.11	4
13	1291.50	1273.58	1274.56	646.30	Q	362.20	344.21	345.19	181.60	3
14	1378.62	1360.61	1361.60	689.81	S	234.14	216.13	217.12	117.57	2
15					K	147.11	129.10	130.09	74.06	1

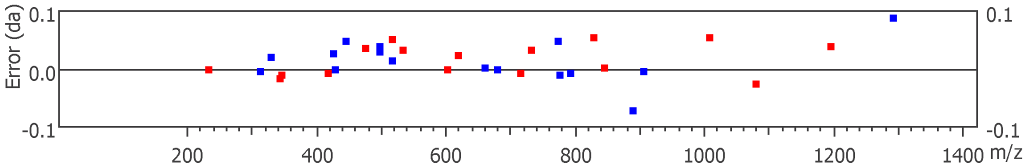

156226146

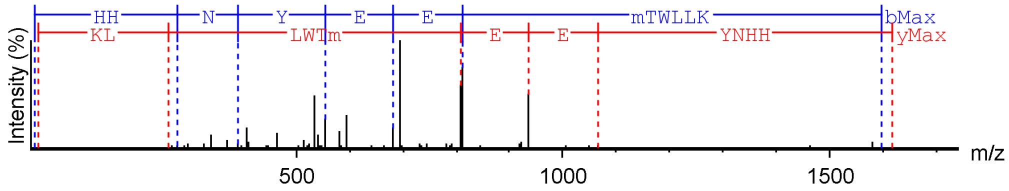

#	b	b-H2O	b-NH3	b (2+)	Seq	y	y-H2O	y-NH3	y (2+)	#
1	138.07	120.06	121.04	69.53	H					12
2	275.11	257.12	258.10	138.06	H	1479.69	1461.68	1462.67	740.32	11
3	389.16	371.14	372.14	195.08	N	1342.63	1324.62	1325.61	671.82	10
4	552.20	534.22	535.20	276.62	Y	1228.59	1210.58	1211.56	614.80	9
5	681.25	663.26	664.25	341.13	E	1065.46	1047.46	1048.50	533.26	8
6	810.30	792.32	793.29	405.66	E	936.53	918.57	919.46	468.75	7
7	957.35	939.34	940.33	479.18	M(+15.99)	807.46	789.47	790.42	404.22	6
8	1058.40	1040.39	1041.37	529.70	T	660.41	642.40	643.36	330.70	5
9	1244.48	1226.47	1227.45	622.74	W	559.36	541.28	542.33	280.18	4
10	1357.56	1339.55	1340.54	679.29	L	373.28	355.27	356.25	187.09	3
11	1470.65	1452.64	1453.62	735.78	L	260.20	242.19	243.12	130.60	2
12					K	147.11	129.10	130.09	74.06	1

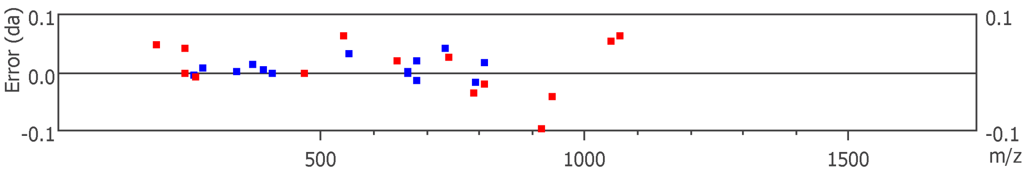

221112215

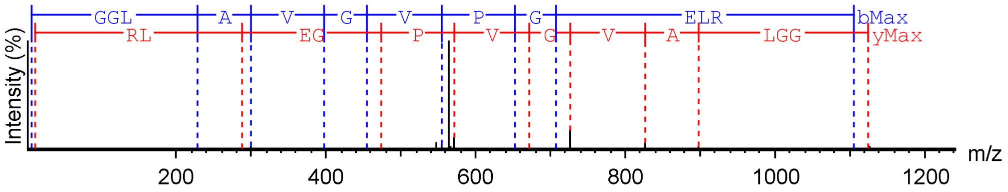

#	b	b-H2O	b-NH3	b (2+)	Seq	y	y-H2O	y-NH3	y (2+)	#
1	58.03	40.02	41.00	29.51	G					12
2	115.05	97.04	98.02	58.03	G	1067.62	1049.61	1050.59	534.31	11
3	228.14	210.12	211.11	114.57	L	1010.60	992.59	993.57	505.80	10
4	299.15	281.16	282.17	150.09	A	897.52	879.50	880.49	449.26	9
5	398.22	380.23	381.21	199.62	V	826.45	808.47	809.48	413.74	8
6	455.24	437.26	438.23	228.13	G	727.39	709.40	710.38	364.20	7
7	554.29	536.36	537.29	277.67	V	670.35	652.38	653.36	335.69	6
8	651.40	633.43	634.36	326.19	P	571.30	553.28	554.29	286.16	5
9	708.32	690.37	691.38	354.70	G	474.23	456.26	457.24	237.63	4
10	837.45	819.44	820.42	419.22	E	417.25	399.24	400.22	209.12	3
11	950.53	932.52	933.50	475.77	L	288.18	270.19	271.18	144.60	2
12					R	175.12	157.11	158.09	88.06	1

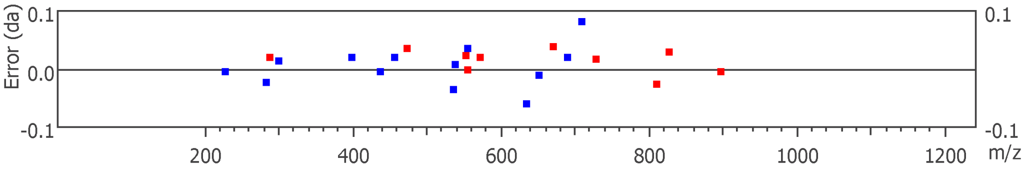

219231428

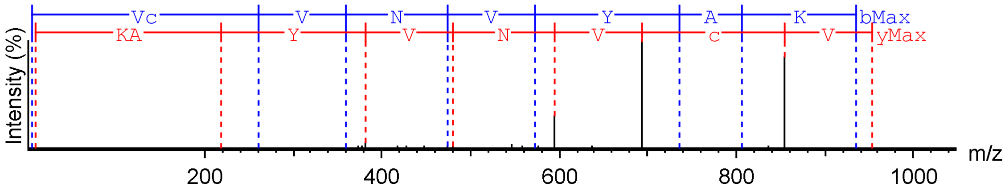

#	b	b-H2O	b-NH3	b (2+)	Seq	y	y-H2O	y-NH3	y (2+)	#
1	100.08	82.07	83.05	50.54	V					8
2	260.09	242.10	243.10	130.55	C(+57.02)	853.41	835.32	836.41	427.21	7
3	359.16	341.17	342.15	180.09	V	693.38	675.38	676.29	347.20	6
4	473.23	455.21	456.14	237.11	N	594.31	576.31	577.28	297.66	5
5	572.25	554.28	555.26	286.64	V	480.27	462.27	463.25	240.64	4
6	735.29	717.34	718.32	368.17	Y	381.20	363.21	364.19	191.11	3
7	806.40	788.38	789.42	403.69	A	218.16	200.14	201.12	109.57	2
8					K	147.11	129.10	130.09	74.06	1

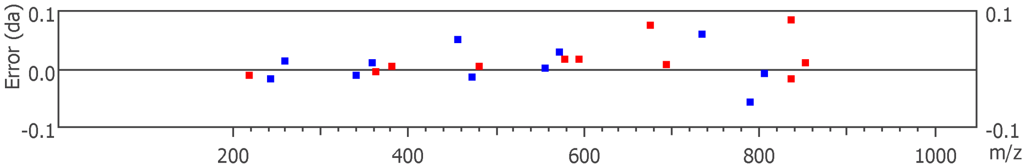

223649562

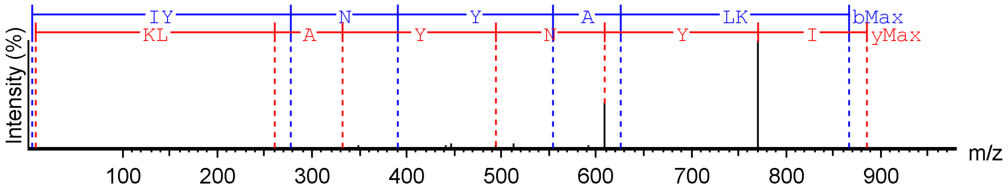

#	b	b-H2O	b-NH3	b (2+)	Seq	y	y-H2O	y-NH3	y (2+)	#
1	114.09	96.08	97.06	57.55	I					7
2	277.14	259.14	260.18	139.08	Y	771.39	753.39	754.37	386.20	6
3	391.20	373.19	374.17	196.10	N	608.32	590.30	591.29	304.67	5
4	554.21	536.25	537.23	277.63	Y	494.31	476.29	477.27	247.65	4
5	625.28	607.30	608.32	313.15	A	331.23	313.22	314.21	166.12	3
6	738.38	720.37	721.36	369.69	L	260.18	242.19	243.17	130.60	2
7					K	147.11	129.10	130.09	74.06	1

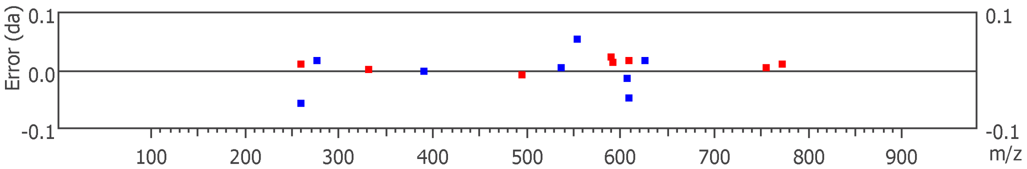

Supplement: Information S6 — Annotated single peptide identifications. Annotated spectra, ion tables and error plots of all proteins identified by a single peptide during homology searches using PEAKS. (PDF) [file pone.0047866.s006.pdf]
